# Explanatory factors of post-traumatic distress and burnout among hospital staff 6 months after Hurricane Irma in Saint-Martin and Saint-Barthelemy

**DOI:** 10.1371/journal.pone.0229246

**Published:** 2020-03-10

**Authors:** Damien Metregiste, Denis Boucaud-Maitre, Lyderic Aubert, Lazare Noubou, Louis Jehel

**Affiliations:** 1 Service des Urgences, Centre hospitalo-Universitaire de Martinique, Fort de France, France; 2 Direction de la Recherche Clinique et de l’Innovation, Centre hospitalo-Universitaire de Guadeloupe, Point-à-Pitre, France; 3 CIRE Antilles, Santé-Publique France, Pointe-à-Pitre, Guadeloupe, France; 4 Centre Hospitalier de Saint Martin et Saint Barthélémy, Service des Urgences et du SMUR, Commission de Qualité et de la Sécurité des Soins, Marigot, France; 5 Service de Psychiatrie, Centre hospitalo-Universitaire de Martinique, Fort de France, Université des Antilles-Guyane, INSERM, Fort de France, France; 6 Président de la Commission Formation et Vie Universitaire pôle formation de la Martinique, Université des Antilles et de la Guyane, Fort de France, France; 7 Centre de recherche en Santé Mentale et Santé Publique, INSERM U1176, Paris, France; Monash University, AUSTRALIA

## Abstract

**Background:**

In September 2017, the Hurricane Irma devastated the islands of Saint-Martin and Saint-Barthelemy (French West Indies). This was a particularly distressing time for the local healthcare staff in charge of rescuing the population. The aim of this study was to identify the explanatory factors of post-traumatic distress and burnout in hospital staff.

**Methods:**

An anonymous questionnaire was sent to all 509 hospital workers of Saint-Martin and Saint-Barthelemy. Post-traumatic distress and burnout was assessed using the Post-Traumatic Stress Disorder Checklist (PCL-S) and Copenhagen Burnout Inventory (CBI) scales. Bivariate and multivariate analyses were used to determine the explanatory variables for these two psychological disorders.

**Results:**

Two hundred and sixty-two questionnaires were completed (response rate of 51.7%). The explanatory factors of post-traumatic distress were female gender (OR = 12.93, 95% CI: 2.70–232.10), electricity shortages (OR = 2.92, 95% CI: 1.13–8.19) and home damage (OR = 1.16, 95% CI [1.02–1.33]). In parallel, the explanatory factors of burnout were post-traumatic distress (OR: 10.42, 95% CI: 4.72–25.58), female gender (OR = 2,41, 95% CI: 1.24–5.02) and paramedical staff (OR = 2,53, 95% CI: 1.15–6.21). In the multivariate analysis, only burnout was significantly associated with post-traumatic distress (OR = 9.26, 95% CI: 4.11–23.14).

**Conclusions:**

Six months after Irma, post-traumatic distress among hospital staff was strongly linked to burnout. This study revealed the lack of electricity as a new factor related to post-traumatic distress. It also suggested that psychological intervention should be strengthened.

## Introduction

**Fig 1:**
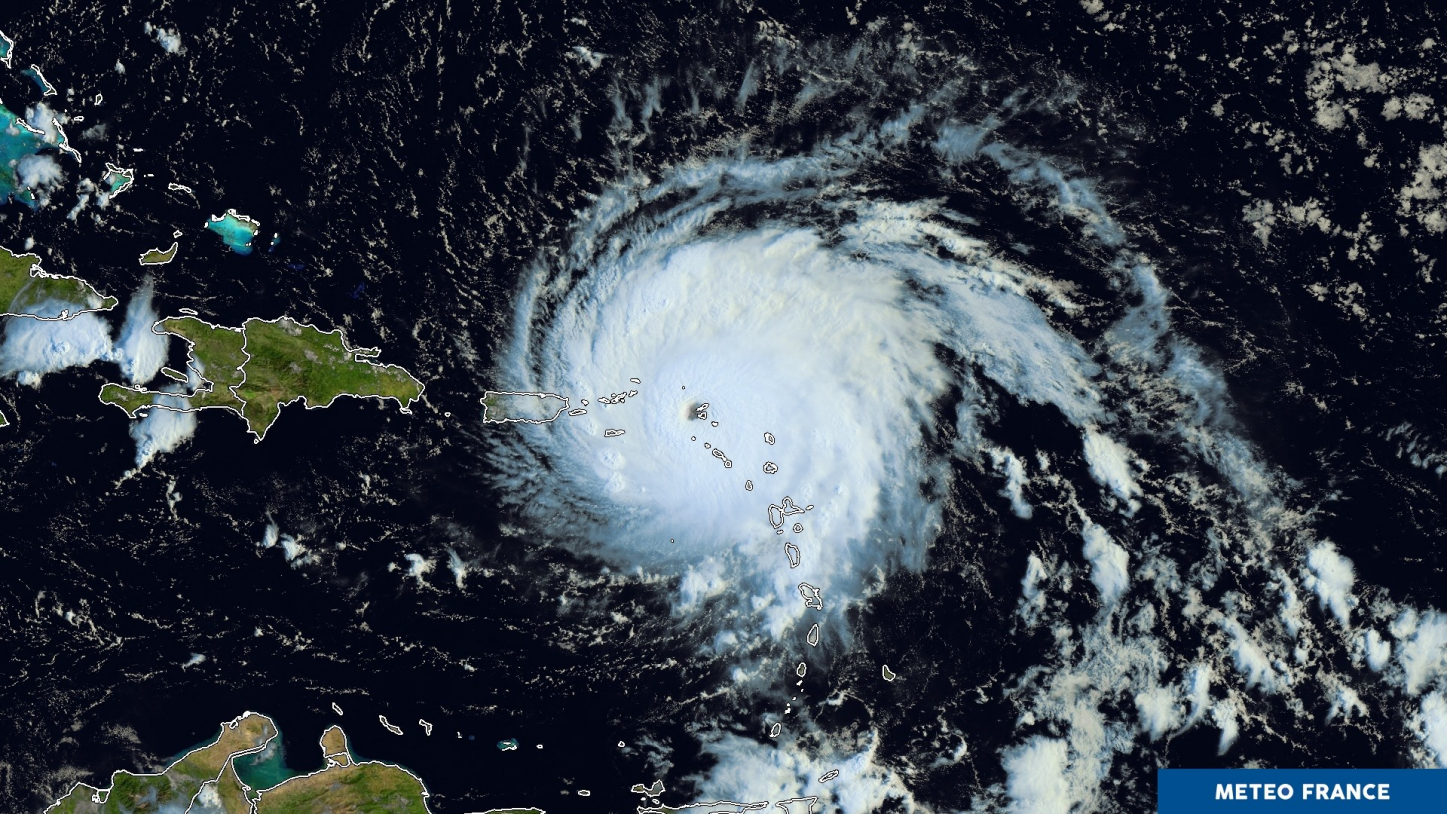
Saint-Martin in the eye of hurricane Irma– 6 September 2017. Satellite image © Météo-France.

Irma was one of the most powerful hurricanes ever recorded in the Atlantic Ocean. It emerged from a tropical depression off the West African coast on August 27, 2017, which gradually strengthened to stage 5 on the Saffir-Sympson scale on 4 September. When the eye of the hurricane reached the islands of Saint-Martin and Saint-Barthelemy on the morning of September 6, average winds were measured at 159 kt (183 mph—295 km/h) and sea level rose abruptly by more than 3 meters, with waves of 4 to 9 meters [1] (Fig 1). The immediate damage was considerable, depriving the whole population of electricity, clean water, communication systems and airport and maritime routes for disaster recovery response. This isolation lasted several days because of the uncertain trajectory of hurricane José near the two islands on September 9 [2]. In total, the official toll of Irma's passage over Saint-Martin and Saint-Barthelemy was 11 fatalities, 95% of homes and infrastructure affected, 60% of damaged buildings made uninhabitable and an estimated 3.5 billion euros cost [3].

The emotional distress caused by hurricane Irma and the prolonged period of exposure to stress that followed, may have led to longer-term psychological disorders, including the emergence of post-traumatic distress (PTD). PTD may develop when a person has lived or has been repeatedly exposed to a traumatic and violent event that threatens physical integrity or result in death [4]. Hospital staff, who always are among first-responders when a traumatic event occurs, are particularly exposed to a medium or long-term risk of post-traumatic distress, whether in the event of a hurricane [5] or a terrorist attack for example [6,7]. However, post-traumatic distress is not the only impact on the mental health of hospital staff. In Saint-Martin and Saint-Barthelemy, the consequences of the hurricane led to a prolonged deterioration of working conditions (significant increase in workload, absenteeism, lack of equipment, etc.), which, combined with the slow return to normal life (housing, electricity, water, etc.), were likely to lead to staff burnout. In addition, managing traumatized patients can be emotionally difficult and stressful for therapists and caregivers, which can also lead to vicarious trauma. The accumulation of a traumatic event on the one hand and high workload on the other may trigger worrying psychological suffering that could in turn lead to serious illnesses.

In this study, we tried to evaluate the impact of Irma on the mental health of hospital staff in Saint-Martin and Saint-Barthelemy, two islands particularly devastated by the hurricane. First, the aim of this study was to identify the explanatory factors of post-traumatic distress and burnout in hospital staff six months after the event. Improving our understanding may help to optimize intervention strategies and prevent post-traumatic distress and burnout post-disaster. Second, our objective was to evaluate the psychological impact that Irma had had on hospital staff. Therefore, we estimated the prevalence of post-traumatic distress and Burnout, but also the consequences of the event on work at the hospital.

## Materials and methods

### Participants and procedure

On February 27, 2018, an e-mail was sent to all hospital staff of Saint-Martin and Saint-Barthelemy present on the date of the hurricane, i.e. 416 and 91 staff respectively. Professional categories were classified as follows: medical staff (doctors, surgeons, pharmacists, residents and midwives) paramedical staff (nurse, head nurse, surgical nurse, nurse anaesthetist, auxiliary nurse, child nurses, child care assistants, psychologists, radiographer and stretcher bearers) and non-clinical staff (hospital managers, administrative assistants, social workers, secretaries, receptionists, computer scientists, technicians, hospital engineers, catering staff, cleaners, workmen). Staff outside the department, on leave or sick leave were also included, as were employees who left their jobs following the hurricane (contacted on their personal e-mail address). Workers who left the hospital before the hurricane and those who arrived at the end of the storm were excluded from the study. After explaining the purpose of the study and informing them of their right to refuse to participate, staff were invited to complete an anonymous questionnaire either online or in paper form with ballot boxes available in each department. The closing date of the investigation was March 11, 2018.

### Measuring instruments

The questionnaire collected the following information: age, gender, occupation in hospital, family status, number of children, personal property and home damage (scale from one to ten with 1 reflecting a minimum degree of damage and 10 the maximum), water and electricity shortages at home, telephone or internet outage, physical damage, theft, assault, work stoppages, psychological care, use of anxiolytics since Irma, length of time before returning to initial accommodation, intention to leave one's job or region. In addition, hospital staff could answer yes or no to the question of whether they thought recent events had affected the quality of their work.

Standardized instruments for measuring post-traumatic distress and burnout were used:

The Post-traumatic stress disorder Checklist Scale—Specific version (PCL-S) was used to determine the presence of post-traumatic distress over the past month [8]. It was translated into French and validated in 2003 for post-traumatic distress screening and follow-up [9]. PCL-S has good psychometric properties [10] and the Cronbach’s alpha value in our sample was 0.93. The 3 dimensions of the scale are "intrusion", "avoidance" and "hyperarousal" via 17 items rated from 1 to 5 (score between 17 and 85). In accordance with the recommendations, the cut-off for the diagnosis of post-traumatic distress has been set for a threshold value greater than or equal to 44 at which point it was considered highly sensitive and specific [11].

The Copenhagen Burnout Inventory (CBI) was used to assess burnout. The CBI was proposed in 2005 in front of the Maslach Burnout Inventory (MBI) limits [12]. It is intended for a general population and not exclusively for employees in human service like the MBI. Although its use remains limited in the literature, it was useful in this study to evaluate all hospital staff, both clinical and non-clinical. The CBI is based on the evaluation of the central component of burnout: "exhaustion". It is divided into 3 scales that can be used independently. They measure: personal burnout, work burnout and relationship burnout. In this study, we evaluated work-related burnout, which is defined as a state of prolonged physical and psychological suffering perceived as work-related. The questionnaire was translated and validated into French [13]. It has good psychometric properties and the Cronbach’s alpha value in our sample was 0.91. The inventory included 7 items rated from 0 to 4 (score between 0 and 28). Score higher than 14 (50% of the maximum score) was considered a diagnosis of burnout [14].

### Data analysis

Quantitative variables were expressed as mean ± standard deviation, categorical variables as percentage. The internal consistency of each scale was assessed by the Cronbach’s alpha coefficient. Bivariate analyses were conducted by logistic regression to investigate the relationship between the presence or absence of post-traumatic distress or burnout with each of the variables of interest (for post-traumatic distress: Hospital, work status, age, gender, marital status, children, tenant/owner, years in Saint-Marteen/Saint-Barthélémy, power outage, water shortage, internet outage, telephone outage, theft, assault, injury, psychological support, back home<1 month, and home damage. For burnout: Hospital, work status, age, gender, marital status, children, tenant/owner, years in Saint-Marteen/Saint-Barthélémy and post-traumatic distress). The multivariate logistic analysis on the post-traumatic distress included the variables whose bivariate analysis had shown a threshold of significance lower than 10% and a step-by-step model was retained. Effect sizes of the associations between the independents and dependent variables were expressed as odds ratios (ORs; i.e., exponential coefficients of independent variables in the logistic regression model).The Spearman correlation coefficient and scatter plots were used to analyse the relationship between the PCL-S and CBI scores. Missing data for PCL-S and CBI items were imputed by the median, given the small number of missing data in each of the questionnaires (0.4% and 0.9% respectively). For p-values, a threshold of 0.05 was considered as an indicator of significance. All analyses were performed with Rv.3.0.2.

### Ethical considerations

The study protocol was reviewed and validated by the Research Ethics Board of St Martin and St Barthelemy Hospitals. Authorization was obtained from the National Commission on Information Technology and Liberties (CNIL: number 2166486). A note for participants on the objectives of this study was included at the beginning of the questionnaire. Staff participation was on a voluntary basis. The data were kept confidential and secure, and only members of the research team had access to the data.

## Results

### Population

Two hundred and sixty-two questionnaires were completed out of a total of 509 questionnaires sent. Our sample was comparable across all subgroups except for non-clinical staff who were slightly under-represented. The response rate was 51.7%. In total 53.6% of women in our study population completed the questionnaire. The response rate by professional category was 51.3% for medical staff, 56.7% for paramedical staff and 36.8% for non-clinicals (Table 1).

**Table 1:** Representativeness of participants.

	Staff*	Sample	Response rate
**GENDER**			
• Women	379	203	53,6%
• Men	128	59	46,1%
AGE**			
• ≤ 40 years old	260	129	49,6%
• > 40 years old	247	127	51,4%
**PROFESSIONAL CATEGORY**			
• Medical staff	76	39	51,3%
• Paramedical staff	314	178	56,7%
• Non-clinical staff	117	45	38,5%
WORK LOCATION***			
• Saint-Martin hospital	416	203	48,8%
• Saint-Barthelemy hospital	91	57	62,6%
**TOTAL**	**507**	**262**	**51,7%**

* Staff present at the date of Irma's passage

** All responders did not indicate their age

*** Two agents working in both hospitals have been removed.

One hundred and thirty-nine (53.0%) people responded online and 123 (47.0%) responded on paper. Respondents were on average 41.0 ± 11.1 years old and had lived on these islands for a long time (14.6 ± 13.0 years). They were overwhelmingly women (n = 203, 77.5% versus 59 men, 22.5%). A majority of respondents lived in couples (64%), with children (60.1%), rented houses or apartments (64.1%). At the time of the hurricane, 184 respondents were in Saint-Martin (71.3%), 54 in Saint-Barthelemy (20.9%) and 20 (7.8%) outside the territory. They were mostly at home (53.6%), in hospital (27.0%) or with friends or neighbors (15.5%). In the days following Irma, 8.9% of respondents were victims of theft, 8.4% of injuries and 2.3% of assaults. On a scale of 1 to 10, respondents rated their home as 5.0 ± 2.7 damaged and their personal property as 4.9 ± 2.8 damaged. One quarter (24.2%) were unable to return to their homes within a month of the hurricane. In addition, 24.9% of respondents suffered power outage at home for more than a month, 27.7% had no telephone, 37.9% had no water and 52.6% had no internet access. Almost a third of the respondents (32.4%) reported having consulted the emergency medical-psychological unit, 29.5% had had work stoppages and 21.9% had used or increased their consumption of anxiolytics since Irma's visit (Table 2).

**Table 2:** Bivariate and multivariate analysis: Explanatory variables associated with post-traumatic distress.

			Bivariate analysis		Multivariate analysis	
	Total	PTD+	p-value	OR (95% CI)	p-value	OR (95% CI)
**Hospital**						
Saint-Martin	203 (78.1%)	33 (16.3%)	p = 0.164	2.02 [0.81–6.13]		
Saint-Barthelemy	57 (21.9%)	5 (8.9%)		1.00		
**Work status**						
Medical staff	39 (16.5%)	2 (5.1%)	p = 0.079	1.00		
Paramedical staff	178 (68.5%)	30 (16.9%)		3.75 [1.06–23.83]		
Non-clinical staff	43 (15.0%)	6 (14.0%)		3.00 [0.64–21.39]		
**Age**						
≤ 40	129 (50.4%)	14 (10.8%)	p = 0.14	1.00		
> 40	127 (49.6%)	22 (17.3%)		1.72 [0.84–3.61]		
**Gender**						
Male	59 (22.5%)	1 (1.7%)	p = 0.012	1.00	0.014	1.00
Female	203 (77.5%)	37 (18.2%)		12.93 [2.70–232.10]		15.82 [2.62–31.88]
**Marital status**						
In relationship	167 (64.0%)	23 (13.8%)	p = 0.651	1.00		
Single	94 (36.0%)	15 (16.0%)		1.19 [0.58–2.19]		
**Children**						
Yes	152 (60.1%)	27 (17.8%)	p = 0.087	1.97 [0.93–4.45]		
No	101 (39.9%)	10 (9.9%)		1.00		
**Tenant/Owner**						
Tenant	168 (64.1%)	19 (11.3%)	p = 0.052	1.00	0.017	2.86 [1.22–6.89]
Homeowner	94 (35.9%)	19 (20.2%)		1.99 [0.99–3.99]		1.00
**Years in St-Martin /St-Barthelemy**						
< 3 years	59 (24.2%)	5 (8.5%)	p = 0.147	1.00		
> 3 years	185 (75.8%)	30 (16.2%)		2.09 [0.83–6.37]		
**Power outage**						
< 1 week	80 (31.1%)	7 (8.7%)	p = 0.029	1.00		
[1 week-1 month]	113 (44.0%)	17 (15.0%)		1.85 [0.75–4.99]		
> 1 month	64 (24.9%)	14 (21.9%)		2.92 [1.13–8.19]		
**Water shortage**						
< 1 week	45 (18.1%)	5 (11.1%)	p = 0.088	1.00		
[1 week-1 month]	102 (44.0%)	12 (11.8%)		0.99 [0.34–3.28]		
> 1 month	94 (37.9%)	19 (20.2%)		2.03 [0.75–6.47]		
**Internet outage**						
< 1 week	48 (19.6%)	5 (10.4%)	p = 0.347	1.00		
[1 week-1 month]	68 (27.8%)	10 (14.7%)		1.48 [0.49–5.05]		
> 1 month	129 (52.6%)	21 (16.3%)		1.67 [0.63–5.26]		
**Telephone outage**						
< 1 week	109 (42.6%)	15 (13.8%)	p = 0.453	1.00		
[1 week-1 month]	76 (29.7%)	9 (11.8%)		0.84 [0.33–2.00]		
> 1 month	71 (27.7%)	13 (18.3%)		1.40 [0.61–3.17]		
**Theft**						
Yes	23 (8.9%)	6 (26.1%)	p = 0.098	2.33 [0.79–6.11]	0.056	3.73 [0.92–14.44]
No	236 (91.1%)	31 (13.1%)		1.00		1.00
**Assault**						
Yes	6 (2.3%)	2 (33.3%)	p = 0.209	3.04 [0.41–16.18]		
No	253 (97.7%)	36(14.2%)		1.00		
**Injury**						
Yes	22 (8.4%)	6 (27.7%)	p = 0.085	2.42 [0.82–6.39]		
No	239 (91.6%)	32 (13.4%)		1.00		
**Psychological support**						
Yes	85 (32.4%)	16 (18.8%)	p = 0.172	1.63 [0.80–3.29]		
No	177 (67.6%)	22 (12.4%)		1.00		
**Back home < 1 month**						
Yes	194 (75.8%)	27 (13.9%)	p = 0.667	1.00		
No	62 (24.2%)	10 (16.1%)		1.19 [0.52–2.55]		
**Home damage**						
N	253	5.0 **±** 2.7	p = 0.027			
Mean ± SD	5.0 **±** 2.7	6.0 **±** 2.6		1.16 [1.02–1.33]		

### Explanatory factors of post-traumatic distress

**Fig 2:**
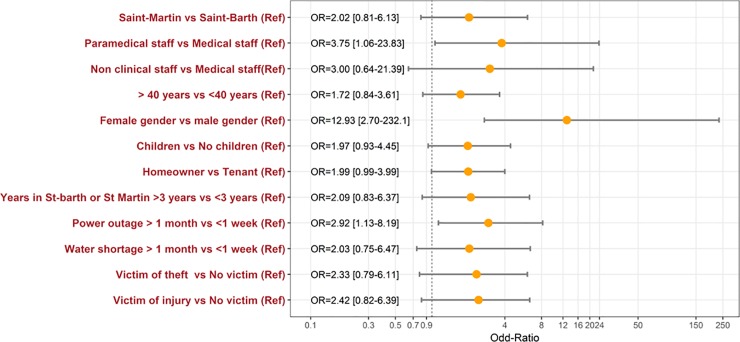
Odds‐ratio of explanatory factors for post-traumatic distress (bivariate analysis with p<0.2).

Six months after the hurricane, 14.5% (95% CI: 10.1–18.9) of respondents (n = 38) had post-traumatic distress. Thirty-seven of 38 respondents with post-traumatic distress were female. Bivariate analyses (Table 2 and Fig 2) showed that the explanatory factors for having post-traumatic distress at 6 months were: female gender (OR = 12.93, 95% CI: 2.70–232.10), home damage (6.0 ± 2.6 versus 4.8 ± 2.7; p = 0.027), and lack of electricity over one month (OR = 2.92, 95% CI: 1.13–8.19) (p = 0.029) especially when it lasts longer than one month. Post-traumatic distress was also observed more often in Saint-Martin than in Saint-Barthelemy (16.3% versus 8.9%, p = 0.164), among paramedical staff (16.9% versus 5.1% among medical staff), among responders with children (OR = 1.97, 95% CI:0.93–4.45; p = 0.087), among responders who had water cuts longer than 1 month (20.2% versus 11.1% for those who had cuts of less than a week), among those who had been victims of theft (OR = 2.33, 95% CI: 0.79–6.11; p = 0.098) or injury (27.7% versus 13.4%, p = 0.085). Having received psychological intervention with the CUMP (emergency medico-psychological unit) didn't appear as protective factor of post-traumatic distress (18.8% against 12.4%; p = 0.172). Multivariate analysis (Table 2) suggested that female gender (OR: 15.82, 95% CI: 2.62–31.88; p = 0.014) and homeowners (2.86, 95% CI: 1.22–6.89; p = 0.017) were associated with post-traumatic distress.

### Explanatory factors of burnout

According to the CBI scale (score ≥ 50%), the prevalence of exhaustion syndrome was 34.1% [95% CI: 28.2–40.0]. Bivariate analyses (Table 3) indicated that burnout was associated with paramedical status (OR = 2.53, 95% CI: 1.15–6.21; compared to medical staff), gender (37.9% among women versus 20.3% among men; p = 0.013) and post-traumatic distress (78.9% versus 26.3%, p<0.001). Multivariate analysis (Table 3) suggested that only post-traumatic distress (OR = 9.26, 95% CI: 4.11–23.14; p = 0.007) was associated with burnout. (Spearman correlation coefficient at 0.51, p<0.001) The Spearman correlation coefficient between post-traumatic distress and burnout was 0.51 (p<0.001).

**Table 3:** Bivariate and multivariate analysis: Explanatory variables associated with burnout.

			Bivariate analysis		Multivariate analysis	
	Total	Burnout	p-value	OR (95% CI)	p-value	OR (95% CI)
**Hospital**						
Saint-Martin	203 (78.1%)	69 (34.0%)	p = 0.810	1.00		
Saint-Barthelemy	57 (21.9%)	20 (35.1%)		1.08 [0.57–1.99]		
**Work status**						
Medical staff	39 (16.5%)	8 (20.5%)	p = 0.029	1.00		
Paramedical staff	178 (68.5%)	70 (39.3%)		2.53 [1.15–6.21]		
Non-clinical staff	43 (15.0%)	11 (25.6%)		1.33 [0.48–3.86]		
**Age**						
≤ 40	129 (50.4%)	48 (37.2%)	p = 0.43	1.22 [0.73–2.06]		
> 40	127 (49.6%)	41 (32.3%)		1.00		
**Gender**						
Male	59 (22.5%)	12 (20.3%)	p = 0.013	1.00		
Female	203 (77.5%)	77 (37.9%)		2.41 [1.24–5.02]		
**Marital status**						
In relationship	167 (64.0%)	61 (36.5%)	p = 0.256	1.37 [0.80–2.38]		
Single	94 (36.0%)	28 (29.8%)		1.00		
**Children**						
Yes	152 (60.1%)	52 (34.2%)	p = 0.772	1.08 [0.64–1.86]		
No	101 (39.9%)	33 (32.7%)		1.00		
**Tenant/Owner**						
Tenant	168 (64.1%)	56 (33.3%)	p = 0.726	1.00		
Homeowner	94 (35.9%)	33 (35.1%)		1.10 [0.64–1.87]		
**Years in St-Martin/St-Barthelemy**						
< 3 years	59 (24.2%)	18 (30.5%)	p = 0.597	1.00		
> 3 years	185 (75.8%)	63 (34.1%)		1.19 [0.64–2.27]		
**PTD**						
Yes	38 (14.5%)	30 (78.9%)	p<0.001	10.42 [4.72–25.58]	0.003	9.26 [4.11–23.14]
No	224 (85.5%)	59 (26.3%)		1.00		1.00

### Impact on work activity

Six months after the hurricane, bivariate analyses (Table 4) reveals that post-traumatic distress and burnout were associated with sick leaves (OR = 2.84, 95% CI:1.40–5.76; and OR = 3.47, 95% CI:2.00–6.10), consumption of anxiolytics (OR = 2.94, 95% CI:1.39–6.11; and OR = 2.29, 95% CI: 1.25–4.18) and intention to change job (OR = 3.14, 95% CI: 1.48–6.54; and OR = 6.80, 95% CI: 3.56–13.54). People with high levels of post-traumatic distress or burnout reported that their work quality was reduced. After Irma, the perception of the quality of work was affected in both respondent victims of post-traumatic distress (OR = 3.99, 95% CI:1.90–9.03) and burnout (OR = 3.77, 95% CI:2.20–6.54). Indeed, 73% of agents suffering from post-traumatic distress, and 65.9% of those with burnout, felt that they were doing a poor-quality job after Irma. Burnout (OR = 2.75, 95% CI: 1.59–4.80) but not post-traumatic distress (OR = 1.94, 95% CI:0.94–3.93) were associated with intention to leave the island.

**Table 4:** Association between post-traumatic distress, burnout and post-Irma issues.

	Total	PTD +	p	OR [IC95%]	Burnout +	p	OR [IC95%]
**Sick leave**							
Yes	77 (29.5%)	19 (24.7%)	p = 0.006	2.84 [1.40–5.76]	42 (54.5%)	p<0.001	3.47 [2.00–6.10]
No	184 (70.5%)	19 (10.3%)		1.00	47 (25.5%)		1.00
**Perception of the quality of work**							
Affected	117 (45.0%)	27 (73.0%)	p<0.001	3.99 [1.90–9.03]	58 (65.9%)	p<0.001	3.77 [2.20–6.54]
Not affected	143 (55.0%)	10 (13.0%)		1.00	30 (34.1%)		1.00
**Consumption of anxiolytics**							
Yes	57 (21.9%)	15 (26.3%)	p = 0.004	2.94 [1.39–6.11]	28 (49.1%)	p = 0.007	2.29 [1.25–4.18]
No	203 (78.1%)	22 (10.8%)		1.00	60 (29.6%)		1.00
**Intention to change job**							
Yes	53 (20.5%)	15 (28.3%)	p = 0.002	3.14 [1.48–6.54]	37 (28.3%)	p<0.001	6.80 [3.56–13.54]
No	206 (79.5%)	23 (11.2%)		1.00	52 (11.2%)		1.00
**Intention to leave the island**							
Yes	77 (29.4%)	16 (20.8%)	p = 0.066	1.94 [0.94–3.93]	39 (50.6%)	p<0.001	2.75 [1.59–4.80]
No	185 (70.6%)	22 (11.9%)		1.00	50 (27.0%)		1.00

## Discussion

To our knowledge, no epidemiological data have been published concerning psychiatric disorders following Irma. This first study reveals several factors associated with the emergence of post-traumatic distress or burnout among hospital staff in the aftermath of a hurricane. In addition, it allows us to estimate the significant psychological consequences of Irma on hospital staff in Saint-Martin and Saint-Barthelemy.

Explanatory factors associated with the risk of developing post-traumatic distress included gender, home damage and electricity shortages over one month. Women were more likely to develop post-traumatic distress, with 37 of the total 38 cases of post-traumatic distress. This over-risk has already been described previously but to a lesser extent [7,15,16]. Indeed, the literature usually suggests that women are twice as vulnerable to post-traumatic distress as men among victims of potentially traumatic events (all causes combined). In the case of healthcare providers, a meta-analysis published in 2016 also observed that women were more vulnerable [17].

Electricity has become essential. Its lack deeply affects daily life with: difficulties for food storage, cooking, communication. It could also contribute to a feeling of insecurity. In our study, the prolonged power outage was associated with a 2.9-fold increased odds of having post-traumatic distress. This explanatory factor has not been examined in previous research. It may help to take proactive actions if a similar disaster occurs in the future. The link between water shortages and post-traumatic distress revealed by the meta-analysis conducted after Hurricane Katrina [16] could not be confirmed in our study. Nevertheless, a similar trend was observed with an increased risk when water supply was out for more than a month. The training skills have been taken into account as well and we found that paramedical staff had a 3.8-fold higher odds of being affected by post-traumatic distress than medical staff. These results corroborate the observations made by De Stefano [7] on the first-responder teams during the terrorist attacks in Paris, in 2015. Indeed, in this study, health professionals with lower training were more inclined to develop post-traumatic distress symptoms. Finally, there was a lower prevalence, but not significant (p = 0.164), of post-traumatic distress in Saint-Barthelemy compared to Saint-Martin. Although the weather event was identical, Irma’s psychological impact differed from island to island. The reason could be the economic and infrastructural inequalities between them. The absence of riots, theft and the prompt rehabilitation of facilities in Saint-Barthelemy, may as well have contributed to reduce the prevalence of post-traumatic distress among hospital staff present on this island. This study allowed us to assess the prevalence of post-traumatic distress six months after the event. An estimated 14.5% of hospital staff suffered from post-traumatic distress, including 16.3% in Saint-Martin and 8.9% in Saint-Barthelemy and this number is expected to increase slightly over the year [18]. In comparison, studies among adult population exposed to previous hurricanes reported post-traumatic distress prevalence of 9% and 14.5% six months after Mitch (1998)[19] and Sandy (2012)[5] and 17.1% 7 to 19 months after Katrina (2005)[20].

Working environment was particularly stressful for healthcare staff. The hospital was also partially devastated by the hurricane. Capacity was reduced from 88 to 17 beds. Drug stocks was also affected. Thanks to the safety systems, the electrical system was able to continue to operate at a minimum. However, the organization was severely affected with health supplies missing or damaged, and communication systems no longer available. The few staff members who remained in hospital after the hurricane had to manage a massive influx of patients. This deterioration in working conditions combined with an intensification of the work rhythm was responsible for chronic stress that explain why the hurricane led to one third of hospital staff reporting exhaustion burnout. We also focused on the factors that explain burnout in the context of a natural disaster. Gender and professional status were significant factors of burnout. Research on burnout among staff hospital gives contradictory results on several points [21–23]. Our analyses found that paramedical employees had higher odds of developing burnout than doctors. The lower training skills of the former, and their higher exposure to heavily traumatized victims (which triggers compassion fatigue) may explain these differences. According to the literature [24], female gender had a much higher burnout rate (37.9% vs 20.3%). The economical and health consequences of burnout are worrying. The perception of work quality among burnout victims suggests a lower level of care.

More than half of the hospital staff (54.5%) who took sick leave in the six months after the hurricane suffered from burnout. In addition, 50.6% of employees in burnout reported that they planned to leave the island before the next hurricane season and 28.3% reported that they wanted to change jobs. Hurricane season in the North Atlantic Ocean takes place from June 1 to November 30. Hospitals are currently preparing to face new weather disasters and to provide relief to people. However, staff shortages seem to pose difficulties in the aftermath of natural disaster. This challenge should be automatically anticipated and considered as one of the high priorities from the outset. In total, we found that 34.1% of staff had CBI scores in favour of a burnout. However, despite the number of studies on the subject, the literature makes it difficult for us to compare our results, so disparate are the estimates.

The literature suggests a strong association between post-traumatic distress and burnout syndrome [25–27]. We found also that post-traumatic distress appears to be the pre-eminent explanatory factor of exhaustion. It led to a 9.3-fold higher odds of developing burnout. Indeed, professional burnout was observed in 78.9% of post-traumatic distress victims. A worker suffering from post-traumatic distress who continues his or her job despite their pathological condition could therefore be more exposed to daily occupational stress. Nevertheless, it is not excluded that this correlation is reversed: people suffering from burnout would be predisposed to develop post-traumatic distress during a traumatic event. A dedicated study, taking into account the level of exhaustion before a traumatic event, would provide support for this hypothesis. Moreover, we found that post-traumatic distress and burnout were both related to several identical issues (sick leave, work quality, consumption of anxiolytics, intention to leave the island or to change work).

Hospital workers healthcare is one of the priorities and the care of traumatised staff is a challenge that is expected to be faced following a disaster. In French territories, the Emergency Medical and Psychological Unit (CUMP) systematically intervenes when significant psychological repercussions are to be expected following a traumatic event. It plays a central role in the prevention of post-traumatic distress and the organization of Post-Immediate Psychotherapeutic Intervention (IPPI) for all traumatized people [28]. This psychological debriefing is a group work focused on the verbalization of emotions. The scientific literature available to date does not provide any evidence of the effectiveness of debriefing interventions such as IPPI [29]. Nevertheless, IPPI was used after Irma to try to prevent the risk of developing psychotraumatic symptoms. It also identifies high-risk individuals for immediate care. To prevent and detect the occurrence of psychological disorders, the IPPI was conducted by the CUMP between day 2 and day 30. This was designed to be extended to all hospital staff following Irma. Yet, after the cyclone, only one third of the staff had access to the preventive intervention set up by the CUMP. This is an extremely surprising outcome that suggests that hospital staff were unconsciously left out by rescuers, who may have focused their interventions on the local population, and who wrongly believed that caregivers in psychological distress did not need to be treated. The second hypothesis would be that hospital staff would be more reluctant to consult the psychological support unit. Certainly, memory disorders related to peri-traumatic dissociation may cause confusion bias and underestimate the results. This would partly explain why only 18.8% of post-traumatic distress victims recall receiving psychological support. In any case, it would be necessary in the future to identify the reasons for such a low rate of consultations among hospital staff.

This study presages future difficulties if adequate measures are not adopted. Post-traumatic distress affects several dimensions. On a personal level, this could lead to an increased risk of addictive behaviors [30], suicide attempts [31], psychiatric comorbidities [32] and organic pathologies such as coronary heart disease [33,34]. At the organizational level, post-traumatic distress and its consequences on work lead to a growing staff shortage through sick leaves and the exodus of traumatized people. The violence of hurricanes of this magnitude justifies psychological intervention in the short and medium term. As these weather phenomena are likely to recur, measures could usefully be found to better protect all hospital staff. The results of our study were shared with local authorities.

Several limitations must nevertheless be taken into account in the analysis of our results. First, the response rate was 51.7%. This is similar to some studies on post-traumatic distress [7, 19, 26]. Nevertheless, except for the non-clinical group, the sample studied was representative of hospital staff (Table 1). Secondly, the post-traumatic distress and burnout assessment tools were based on self-report questionnaires which, while validated, could not equal the clinical evaluation of a practitioner. Thirdly, six months after the event, it is expected that some of the participants' responses regarding time spent without services (water, electricity, internet and phone network) may have been distorted through a memory bias. Finally, other explanatory factors were not examined (ethnicity, stress perceived), including psychiatric history (co-addiction, depression and other psychiatric disorders before events).

## Conclusion

Six months after Hurricane Irma, post-traumatic distress among hospital staff was strongly linked to burnout and female gender. This study reveals the lack of electricity as a new factor related to post-traumatic distress. In addition, our observations suggested that psychological support, usually systematic in post-disaster periods, had not been sufficient for hospital staff. Yet, the health care of hospital staff is a challenge that must be anticipated. Providing them with medico-psychological support in the wake of a hurricane is a priority. Psychological monitoring of staff, strategies against psychosocial risk factors and staff awareness need to be effectively enforced, in natural disaster-prone areas. In the future, researchers should focus on improving these interventions.

## Supporting information

S1 Data (XLS)

S1 File (PDF)

S2 File (PDF)
