# Supplementary material for: Explanatory factors of post-traumatic distress and burnout among hospital staff 6 months after Hurricane Irma in Saint-Martin and Saint-Barthelemy
Source: PLoS One. 2020 Mar 10;15(3):e0229246. doi: 10.1371/journal.pone.0229246 (PMC7064261; doi:10.1371/journal.pone.0229246)

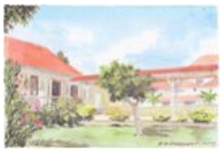

Questionnaire sur l'impact du cyclone IRMA sur le personnel des C.H de Saint Martin et de Saint Barthélémy

Malgré la médiatisation des préjudices causés par le cyclone Irma, il est paradoxal de constater le faible intérêt porté à la santé du personnel hospitalier, sujet tabou et bien peu étudié.
Il semble délicat d'admettre que les garants du système de santé puissent être à leur tour en détresse

Afin de mieux appréhender les conséquences du cyclone Irma sur votre vie personnelle et professionnelle, nous vous invitons à participer à cette enquête.
Cela vous prendra environ 6 minutes. Vos réponses seront anonymes et traitées de manière confidentielle.

Cette étude vise uniquement le personnel sous contrat avec les hôpitaux de Saint Martin et de Saint Barthélémy à la date du passage du cyclone Irma. Merci de ne pas répondre à ce questionnaire si vous n'étiez pas employé par l'hôpital à cette période.

Veillez à ne pas réaliser de doublon avec le formulaire en ligne.

Une fois complété, le formulaire pourra être déposé dans les urnes spécifiques situés dans chaque service.

Merci pour votre participation

I- **Situation personnelle et environnementale**

1- Quel est votre Age : .... ans

2- Quel est votre sexe : M ☐ F ☐

3- Vous travaillez au :

☐ C.H Fleming à Saint Martin
☐ C.H de Bruyn à Saint Barthélémy

4- Quelle est votre profession (emploi au sein de l'hôpital) ? ....

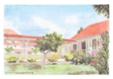

5- Quelle est votre situation maritale et nombre d'enfant :

☐ Célibataire ☐ Marié(e)
☐ En couple ☐ Divorcé(e)
☐ Pacsé(e) Nombre d'enfant: ….

6- Vous êtes (cochez la situation appropriée) :

☐ Locataire
☐ Propriétaire

7- Depuis combien de temps habitez-vous à Saint Martin ou à Saint Barthélémy? ....

II- Dégâts que vous avez subi durant Irma

8- Maison :
(Entourer la case correspondante)

😊	1	2	3	4	5	6	7	8	9	10	😡

9- Bien personnel :

😊	1	2	3	4	5	6	7	8	9	10	😡

10- Combien de temps avez-vous été privé de :

	**moins de 48h**	**moins d'une semaine**	**moins d'un mois**	**plus d'un mois**
Électricité :	☐	☐	☐	☐
Eau :	☐	☐	☐	☐
Téléphone portable :	☐	☐	☐	☐
Internet :	☐	☐	☐	☐

11- Avez-vous été victime de pillage ou de vols ? OUI ☐ NON ☐

12- Avez-vous été blessé pendant le cyclone ? OUI ☐ NON ☐

13- Avez-vous été victime d'agressions physiques : OUI ☐ NON ☐

14- Où étiez-vous durant IRMA ? *plusieurs réponses possibles*

☐ À St Martin
☐ À St Barthélémy
☐ Hors du département

☐ Chez vous
☐ Chez des amis / voisins
☐ Dans un abri anticyclonique
☐ À l'hôpital
☐ Autre

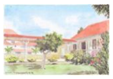

III- Problèmes et symptômes à la suite d’Irma

Veuillez répondre seulement si vous avez été confronté à Irma
Entourez la fréquence à droite de chaque question pour indiquer à quel point vous avez été perturbé par le problème dans **ces 30 derniers jours :** (l'expérience stressante se réfère à Irma)

	**Pas du tout**	**Un peu**	**Par-fois**	**Souvent**	**Très souvent**
15- Êtes-vous perturbé(e) par des souvenirs, des pensées ou des images en relation avec Irma ?	☐	☐	☐	☐	☐
16- Êtes-vous perturbé(e) par des rêves répétés en relation avec Irma ?	☐	☐	☐	☐	☐
17- Avez-vous l’impression de sentir ou d’agir brusquement comme si l’évènement se reproduisait ?	☐	☐	☐	☐	☐
18- Vous sentez-vous bouleversé(e) lorsque quelque chose vous rappelle cet épisode ?	☐	☐	☐	☐	☐
19- Avez-vous des réactions physiques, comme par exemple des battements de cœur, difficultés à respirer, sueurs lorsque quelque chose vous rappelle cet évènement ?	☐	☐	☐	☐	☐
20- Evitez-vous de penser ou de parler de la catastrophe vécue ?	☐	☐	☐	☐	☐
21- Evitez-vous des activités ou des situations parce qu’elles vous rappellent Irma ?	☐	☐	☐	☐	☐
22- Avez-vous des difficultés à vous souvenir de parties importantes de l’expérience stressante ?	☐	☐	☐	☐	☐
23- Avez-vous perdu de l’intérêt pour les activités qui habituellement vous faisaient plaisir ?	☐	☐	☐	☐	☐
24- Vous sentez-vous distant(e) ou coupé(é) des autres personnes?	☐	☐	☐	☐	☐

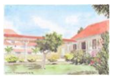

25- Vous sentez-vous émotionnellement anesthésié(e) ou incapable d’avoir des sentiments d’amour pour ceux qui sont proches de vous ?	☐	☐	☐	☐	☐
26- Sentez-vous comme si votre avenir était en quelque sorte raccourci ?	☐	☐	☐	☐	☐
27- Avez-vous des difficultés pour vous endormir ou rester endormi(e) ?	☐	☐	☐	☐	☐
28- Êtes-vous irritable ou avez-vous des bouffées de colère ?	☐	☐	☐	☐	☐
29- Avez-vous des difficultés à vous concentrer ?	☐	☐	☐	☐	☐
30- Êtes-vous en état de super-alarme, sur la défensive, ou sur vos gardes ?	☐	☐	☐	☐	☐
31- Vous sentez-vous énervé(e) ou sursautez-vous facilement ?	☐	☐	☐	☐	☐

IV- Épuisement professionnel ressenti après Irma (Une seule réponse possible)

32 - Mon travail m'épuise à un degré :

◯Très faible ◯Faible ◯Moyen ◯Élevé ◯Très élevé

33 - Mon travail me frustre à un degré :

◯Très faible ◯Faible ◯Moyen ◯Élevé ◯Très élevé

34 - Mon travail est émotionnellement épuisant à un degré :

◯Très faible ◯Faible ◯Moyen ◯Élevé ◯Très élevé

35 - Je me sens vidé(e) à la fin d'une journée de travail :

◯ Jamais ◯Un peu ◯Parfois ◯ Souvent ◯ Tout le temps

36 - En me levant, je me sens déjà épuisé(e) à l'idée d'une autre journée de travail :

◯ Jamais ◯Un peu ◯Parfois ◯ Souvent ◯ Tout le temps

37 - Chaque heure de travail me paraît éprouvante :

◯ Jamais ◯Un peu ◯Parfois ◯ Souvent ◯ Tout le temps

38 - Je manque d'énergie dans les activités de loisir avec ma famille et mes amis :

◯ Jamais ◯Un peu ◯Parfois ◯ Souvent ◯ Tout le temps

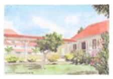

V- **Les complications associées à Irma**

39- Avez-vous bénéficié d’un ou de plusieurs arrêts de travail à la suite d’Irma ?

☐ OUI ☐ NON

o Si oui : -> combien d’arrêts de travail ? ....

-> combien de jours d’arrêt au total ? .... jour(s)

40- Combien de jours de travail avez-vous manqué à cause d’Irma ? (arrêt maladie et autre)

- > …… jour(s)

41- Avez-vous pu revenir habiter dans votre logement dans le mois suivant IRMA ?

☐ OUI ☐ NON

42- Avez-vous pu vous rendre à l’hôpital pour reprendre votre poste, comme prévu sur le planning, dans les jours suivant le cyclone ?

☐ OUI ☐ NON Si non pourquoi ?

☐ Pour vous occuper de votre maison
☐ Pour vous occuper de votre famille
☐ Vous ne vous sentiez pas capable de travailler
☐ Difficultés de locomotion
☐ Hors de l’île
☐ Autre : ....

43- Avez-vous eu un contact avec la cellule d’urgence psychologique (CUMP) présente dans le mois suivant Irma ? (débriefing, intervention collective ou individuelle, consultation ou simple discussion)

☐OUI ☐NON

44- Avez-vous consommé des anxiolytiques depuis Irma (ou majoré votre consommation) ?

☐OUI ☐NON

45- Pensez-vous que les évènements ont pu altérer la qualité de votre travail ?

☐OUI ☐NON

46- Avez-vous ou souhaitez-vous changer de travail depuis Irma ?

☐OUI ☐NON

47- Avez-vous ou souhaitez-vous quitter définitivement l’île ?

☐OUI ☐NON

-> Si oui, quelles sont les raisons de votre départ ?

☐ Économique ☐ Professionnelle ☐ Familiale ☐ Insécurité ☐ Autre :

Merci pour le temps que vous avez consacré à cette enquête

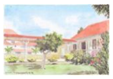

Supplement: S1 File — (PDF) [file pone.0229246.s002.pdf]
