# Supplementary material for: Explanatory factors of post-traumatic distress and burnout among hospital staff 6 months after Hurricane Irma in Saint-Martin and Saint-Barthelemy
Source: PLoS One. 2020 Mar 10;15(3):e0229246. doi: 10.1371/journal.pone.0229246 (PMC7064261; doi:10.1371/journal.pone.0229246)

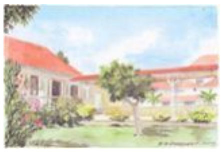

Survey to assess the impact of the cyclone Irma on the hospital staff of Saint Martin and Saint Barthelemy

Despite the media coverage of Irma's damage, it is paradoxical to note the lack of interest in the health of hospital staff, a taboo subject that has not been much studied. It seems difficult to admit that the guarantors of the health system may in turn be in distress

To get a better understanding of the consequences of Hurricane Irma on your personal and professional life we invite you to participate in this survey.

It will take you about 6 minutes. Your answers will be anonymous and processed in a confidential manner.

This study only covers staff under contract with the hospitals of Saint Martin and Saint Barthélémy on the date of Cyclone Irma. Please do not answer this questionnaire if you were not employed by the hospital at that time.

Please, complete this questionnaire only once and be sure to not make doubles with the online form.

Once completed, the questionnaire can be deposited in the specific ballot boxes located in each department.

Thank you for your participation

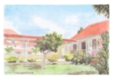

I- Personal and environmental situation

1- How old are you? -> .... years old

2- What is your gender? Men ☐ Female ☐

3- You work at:

☐ Hospital Fleming in Saint Martin
☐ Hospital de Bruyn in Saint Barthélémy

4- What is your profession (employment in the hospital) ? -> ....

5- What is your marital status and number of children?

☐ Single
☐ In couple
☐ PACS (= French Civil solidarity pact)
☐ Married
☐ Divorced
Number of children: ….

6- You are (check the appropriate situation):

☐ Tenant
☐ The owner of the property

7- Since how long do you live in Saint Martin or Saint Barthélémy? -> ....

II- Damage experienced during Irma

8- House:
(Circle the corresponding case)

1	2	3	4	5	6	7	8	9	10

9- Personal property :

1	2	3	4	5	6	7	8	9	10

10- How long have you been missing of :

	**Less than 48 hours**	**Less than a week**	**Less than a month**	**More than a month**
Electricity:	☐	☐	☐	☐
Water :	☐	☐	☐	☐
Mobile phone:	☐	☐	☐	☐
Internet :	☐	☐	☐	☐

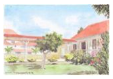

11- Have you been victim of looting or robbery? YES ☐ NO ☐

12- Were you injured during the cyclone? YES ☐ NO ☐

13- Were you physically assaulted in the aftermath of the cyclone? YES ☐ NO ☐

14- Where were you during IRMA? *Several possible answers*

☐ In St Martin
☐ In St Barthélémy
☐ Out of the department

☐ At your home
☐ With friends / neighbours
☐ In a cyclone shelter
☐ At the hospital
☐ Somewhere else

III- Difficulties and symptoms following Irma

Below is a list of problems and complaints that people sometimes have in response to stressful life experiences. Please read each one carefully, then circle one of the numbers to the right to indicate how much you have been bothered by the problem **in the past month.** (The stressful experience refers to Irma)

Please answer only if you experienced **the event Irma.**

	Not at all	A little bit	Moder-ately	Quite a bit	Extremely
15- Repeated disturbing memories, thoughts, or images of Irma's experience?	☐	☐	☐	☐	☐
16- Repeated, disturbing dreams of the stressful experience?	☐	☐	☐	☐	☐
17- Suddenly acting or feeling as if the stressful experience were happening again (as if you were reliving it)?	☐	☐	☐	☐	☐
18- Feeling very upset when something reminded you of the stressful experience?	☐	☐	☐	☐	☐
19- Having physical reactions (e.g., heart pounding, trouble breathing, or sweating) when something reminded you of the stressful experience?	☐	☐	☐	☐	☐

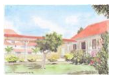

20- Avoiding thinking about or talking about the stressful experience or avoiding having feelings related to it?	☐	☐	☐	☐	☐
21- Avoiding activities or situations because they remind you of the stressful experience?	☐	☐	☐	☐	☐
22- A Trouble remembering important parts of the stressful experience?	☐	☐	☐	☐	☐
23- Loss of interest in activities that you used to enjoy?	☐	☐	☐	☐	☐
24- Feeling distant or cut off from other people?	☐	☐	☐	☐	☐
25- Feeling emotionally numb or being unable to have loving feelings for those close to you?	☐	☐	☐	☐	☐
26- Feeling as if your future will somehow be cut short?	☐	☐	☐	☐	☐
27- Trouble falling or staying asleep?	☐	☐	☐	☐	☐
28- Feeling irritable or having angry outbursts?	☐	☐	☐	☐	☐
29- Having difficulty concentrating?	☐	☐	☐	☐	☐
30- Being “super alert” or watchful or on guard?	☐	☐	☐	☐	☐
31- Feeling jumpy or easily startled?	☐	☐	☐	☐	☐

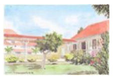

IV- Professional exhaustion felt after Irma (Only one answer by item)

32 - Do you feel burnt out because of your work?
◯To a very low degree ◯To a low degree ◯Somewhat ◯To a high degree ◯To a very high degree

33 - Does your work frustrate you?
◯To a very low degree ◯To a low degree ◯Somewhat ◯To a high degree ◯To a very high degree

34 - Is your work emotionally exhausting?
◯To a very low degree ◯To a low degree ◯Somewhat ◯To a high degree ◯To a very high degree

35 - Do you feel worn out at the end of the working day? a:
◯Never ◯Seldom ◯Sometimes ◯Often ◯Always

36 - Are you exhausted in the morning at the thought of another day at work?
◯Never ◯Seldom ◯Sometimes ◯Often ◯Always

37 - 6. Do you feel that every working hour is tiring for you?
◯Never ◯Seldom ◯Sometimes ◯Often ◯Always

38 - Do you have enough energy for family and friends during leisure time?
◯Never ◯Seldom ◯Sometimes ◯Often ◯Always

V- Complications connected with Irma

39- Have you had one or more work stoppages following Irma?
☐ YES ☐ NO
o If yes : -> how many work stoppages ? ....
-> how many days off in total? .... day(s)

40- How many days of work did you miss because of Irma? (sick leave and other)
- > …… day(s)

41- Were you able to return to your home within one month of Irma?
☐ YES ☐ NO

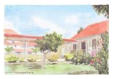

42- Were you able to go to the hospital to take up your shift, as planned, in the days following the cyclone?

☐ YES ☐ NO If no, please explain why not?

- ☐ To take care of your home
- ☐ To take care of your family
- ☐ You didn't feel able to work
- ☐ Difficulties of locomotion
- ☐ Off the island
- ☐ Other : ....

43- Have you had a contact with the psychological emergency unit (CUMP) present in the month following Irma? (debriefing, collective or individual intervention, consultation or simple discussion)

☐ YES ☐ NO

44- Have you taken any anxiolytics since Irma (or increased your consumption)?

☐ YES ☐ NO

45- Do you think that the events may have affected the quality of your work?

☐ YES ☐ NO

46- Have you changed your job since Irma? Or do you plan to?

☐ YES ☐ NO

47- Have you left the island permanently? Or do you intend to?

☐ YES ☐ NO

-> If so, what are the reasons for your departure?

☐ Economic ☐ Professional ☐ Family ☐ Insecurity ☐ Other :…

Thank you for the time you have devoted to this survey

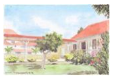

Supplement: S2 File — (PDF) [file pone.0229246.s003.pdf]
